# Design and rationale of the Danish trial of beta-blocker treatment after myocardial infarction without reduced ejection fraction: study protocol for a randomized controlled trial

**DOI:** 10.1186/s13063-020-4214-6

**Published:** 2020-05-23

**Authors:** Anna Meta Dyrvig Kristensen, Ann Bovin, Ann Dorthe Zwisler, Charlotte Cerquira, Christian Torp-Pedersen, Hans Erik Bøtker, Ida Gustafsson, Karsten Tange Veien, Kristian Korsgaard Thomsen, Michael Hecht Olsen, Mogens Lytken Larsen, Olav Wendelboe Nielsen, Per Hildebrandt, Sussie Foghmar, Svend Eggert Jensen, Theis Lange, Thomas Sehested, Tomas Jernberg, Dan Atar, Borja Ibanez, Eva Prescott

**Affiliations:** 1 Department of Cardiology, Bispebjerg University Hospital, Copenhagen, Denmark; 2 Department of Cardiology, Sygehus Lillebælt, Vejle, Denmark; 3 Danish Centre for Rehabilitation and Palliative Care, Odense University Hospital and University of Southern Denmark, Odense, Denmark; 4 The Regional Clinical Quality Development Program, Aarhus, Denmark; 5 Department of Epidemiology and Biostatistics, Aalborg University Hospital, Aalborg, Denmark; 6 Department of Cardiology, Aarhus University Hospital, Aarhus, Denmark; 7 Department of Cardiology, Odense University Hospital, Odense, Denmark; 8 Department of Cardiology, Sydvestjysk Sygehus, Esbjerg, Denmark; 9 Department of Internal Medicine, Holbæk Hospital, Holbæk, Denmark; 10 Department of Cardiology, Aalborg University Hospital, Aalborg, Denmark; 11 Department of Cardiology, Frederiksberg Heart Clinic, Copenhagen, Denmark; 12 Department of Cardiology, Hvidovre-Amager Hospital, Hvidovre, Denmark; 13 Section of Biostatistics, University of Copenhagen, Copenhagen, Denmark; 14 Department of Clinical Sciences, Division of Cardiology, Karolinska Institutet, Danderyd Hospital, Stockholm, Sweden; 15 Department of Cardiology, Oslo University Hospital, Oslo, Norway; 16 Centro Nacional de Investigaciones Cardiovasculares (CNIC) & IIS- Fundación Jiménez Díaz & CIBERCV, Madrid, Spain

**Keywords:** Myocardial infarction, Beta-blocker treatment, Long-term prognosis, Randomized controlled trial

## Abstract

**Background:**

Treatment with beta-blockers is currently recommended after myocardial infarction (MI). The evidence relies on trials conducted decades ago before implementation of revascularization and contemporary medical therapy or in trials enrolling patients with heart failure or reduced left ventricular ejection fraction (LVEF ≤ 40%). Accordingly, the impact of beta-blockers on mortality and morbidity following acute MI in patients without reduced LVEF or heart failure is unclear.

**Methods/design:**

The Danish trial of beta-blocker treatment after myocardial infarction without reduced ejection fraction (DANBLOCK) is a prospective, randomized, controlled, open-label, non-blinded endpoint clinical trial designed to evaluate the efficacy of beta-blocker treatment in post-MI patients in the absence of reduced LVEF or heart failure. We will randomize 3570 patients will be randomized within 14 days of index MI to beta-blocker or control for a minimum of 2 years. The primary endpoint is a composite of all-cause mortality, recurrent MI, acute decompensated heart failure, unstable angina pectoris, or stroke. The primary composite endpoint will be assessed through locally reported and adjudicated endpoints supplemented by linkage to the Danish national registers. A number of secondary endpoints will be investigated including patient reported outcomes and cardiovascular mortality. Data from similar ongoing trials in Norway and Sweden will be pooled to perform an individual patient data meta-analysis.

**Discussion:**

DANBLOCK is a randomized clinical trial investigating the effect of long-term beta-blocker therapy after myocardial infarction in patients without heart failure and reduced LVEF. Results from the trial will add important scientific evidence to inform future clinical guidelines.

**Trial registration:**

Clinicaltrials.gov, NCT03778554. Registered on 19 December 2018.

European Clinical Trials Database, 2018-002699-42, registered on 28 September 2018.

## Background

Beta-blockers have been an essential part of secondary prevention after myocardial infarction (MI) since landmark studies in the 1980s demonstrated a significantly lower mortality associated with beta-blocker treatment [1–3]. The benefits have been attributed to the negative chronotropic and ionotropic features, reducing oxygen demand, and improving coronary diastolic perfusion. Moreover, beta-blockers reduce the sympathetic activation and myocardial sensitivity to fatal arrhythmias [4, 5]. During the last decades reperfusion strategies and improved medical therapy for secondary prevention have substantially improved the prognosis for patients with MI [6]. Introduction of primary percutaneous coronary intervention (PCI) has resulted in increased salvage of myocardium, a smaller ischemic substrate, and a myocardium that is less susceptible to arrhythmias [7]. Hence, long-term oral beta-blocker therapy in patients without reduced left ventricular ejection fraction (LVEF) and heart failure (HF) after MI has been questioned [8, 9].

### Randomized controlled trials evaluating beta-blocker therapy

The beta-blocker trials conducted in the 1980s and 1990s were summarized in a meta-analysis of 82 randomized controlled trials documenting a significantly reduced mortality with beta-blocker therapy after MI [10]. However, the trials included in the meta-analysis were performed before revascularization was standard of care. In a recent meta-analysis comparing randomized controlled trials from the pre-reperfusion and reperfusion era, the mortality benefit was only seen in the pre-reperfusion era [11]. The incidence ratio for mortality in patients with and without beta-blocker treatment after MI in the 12 studies conducted in the reperfusion era was 0.98 (95% confidence interval (CI) 0.92–1.05). In most trials, beta-blockers were given without systematically assessing LVEF, and although the incidence rates of MI and angina pectoris were reduced, there was an increased risk of HF, cardiogenic shock, and drug discontinuation. Analyses were not stratified by LVEF and no trials in the reperfusion era had a follow-up period of more than 1 year. Consequently, it is not possible to extrapolate the results from the meta-analysis to contemporary long-term secondary prevention in patients without HF.

In the reperfusion era only one randomized controlled trial (CAPITAL-RCT) [12] has investigated the long-term efficacy of beta-blockers in patients with LVEF ≥ 40% after MI and successful PCI. The trial included patients with ST-elevation MI (STEMI) and suggested no benefit of carvedilol on a composite outcome (all-cause mortality, MI, hospitalization for HF and acute coronary syndrome) after a median follow up time of 3.9 years. However, the trial was not powered to detect a difference in the treatment effect for the primary endpoint as only 801 patients were randomized.

### Evidence from meta-analyses of observational studies

Given the lack of contemporary randomized controlled trials from the reperfusion era addressing long-term beta-blocker therapy in MI patients without HF, a number of observational and registry studies have addressed the issue and have been summarized in meta-analyses [11, 13–16]. A meta-analysis of seven observational studies from 1996 to 2005 evaluated the benefit of long-term beta-blocker therapy (> 6 months) in STEMI patients with LVEF > 40% who underwent primary PCI [15]. The meta-analysis found long-term beta-blocker therapy to be associated with decreased all-cause mortality (combined hazard ratio 0.79, 95% CI 0.65–0.97). A total of 10,857 patients were included in the analysis and follow-up duration ranged from 6 months to 5.2 years. Another meta-analysis included ten observational studies of patients treated with PCI for either non-ST-elevation MI (NSTEMI) or STEMI with a follow up of at least 3 months. No significant mortality benefit was found in the subgroup of patients with preserved LVEF (relative risk 0.79, 95% CI 0.59–1.07) [16]. Follow-up duration ranged between 6 months and 4 years and a potential efficacy of beta-blocker therapy appeared to gradually disappear > 1 year after MI. A third meta-analysis including a systematic review based on 16 observational studies from 2010 to 2017 found a reduction in all-cause mortality among almost 200,000 post-MI patients without HF (rate ratio 0.74, 95% CI 0.64–0.85). However, publication bias and a small study effect were present. The beneficial effect of beta-blocker therapy disappeared when controlling for bias, suggesting no improved survival with beta-blocker therapy (rate ratio 0.90, 95% CI 0.77–1.04) [13].

Observational studies have thus yielded disparate results and one concern is that they are likely to suffer from confounding by indication as beta-blockers are often avoided in, for example, elderly patients, who have a high burden of comorbidity. In a UK registry-based study including 179,810 MI patients with LVEF > 30%, beta-blockers were prescribed in 95% of the patients. Significant differences in baseline characteristics among the beta-blocker and non-beta-blocker group were evident [17], suggesting that use of beta-blocker therapy is so wide-spread that observational studies are not likely to clarify the value of beta-blocker therapy, even when applying advanced models to adjust for bias. Indeed, this registry-based study found lower mortality rate among patients treated with beta-blockers, but after propensity score and instrumental variable analysis, no difference in survival was found among the two groups.

### Guideline recommendations

Given these uncertainties, contemporary guidelines differ regarding recommendations of beta-blocker therapy. The American College of Cardiology/American Heart Association (ACC/AHA) recommend beta-blocker therapy with a class I recommendation (level of evidence (LOE) B) in patients with STEMI [8], but acknowledge that long-term beta-blocker therapy has not been prospectively addressed. The European Society of Cardiology (ESC) Guidelines for the management of acute MI in patients presenting with STEMI recommend that oral beta-blocker therapy should be considered during hospital stay and continued thereafter in all patients without contraindications with a class IIa recommendation (LOE B) [18]. For NSTEMI patients with preserved LVEF the ACC/AHA guidelines find it reasonable to continue beta-blocker therapy with a class IIa recommendation (LOE C) [19], whereas the ECS 2015 guideline has no recommendations concerning non-ST-elevation acute coronary syndromes [20]. In contrast, the 2013 NICE guideline recommends continuing beta-blocker therapy for at least 1 year after MI in patients without reduced LVEF or HF [21].

### Compliance and side effects

Beta-blockers are generally well tolerated, but side-effects are common and may adversely affect quality of life [1, 2]. Compared with other cardiovascular medications, a lower compliance with beta-blocker treatment is found, suggesting a relation to side effects [22, 23]. However, not all of these side effects are supported by evidence from randomized trials [24].

### Objectives

Given the lack of clear evidence, there has been a call for randomized trials [8, 9, 25, 26]. The objective of the Danish trial of beta-blocker treatment after myocardial infarction without reduced ejection fraction (DANBLOCK) is to evaluate the continued efficacy of long-term beta-blocker therapy after MI in patients without HF. The study hypothesizes that beta-blocker therapy is superior to standard of care without beta-blockers following MI in patients without reduced LVEF (> 40%) and HF receiving contemporary treatment.

## Methods/design

### Trial design

**Fig. 1:**
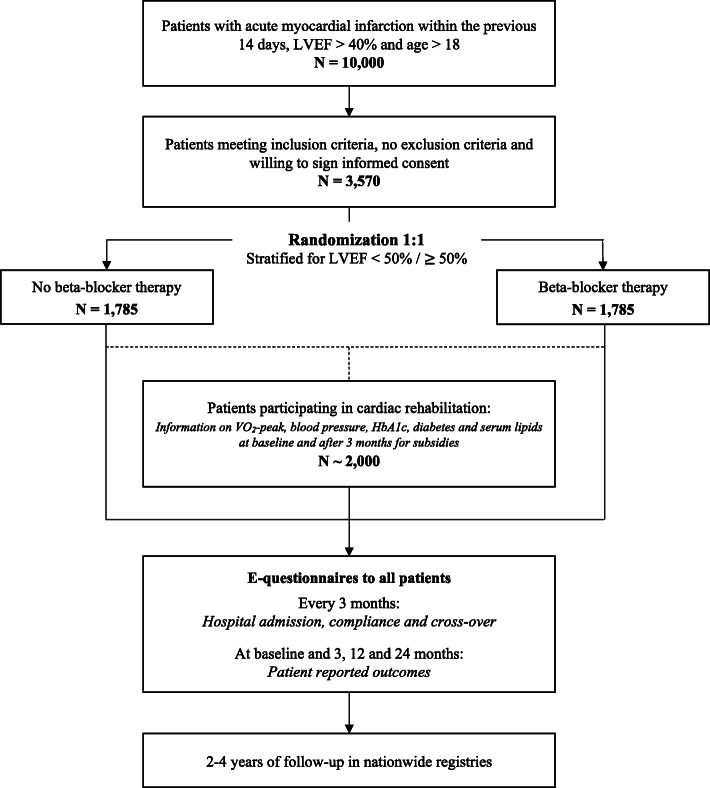
Study overview

DANBLOCK is a Danish multicenter, prospective, randomized, controlled, open-label, non-blinded endpoint clinical trial designed to evaluate the benefits of long-term oral beta-blocker therapy in patients discharged after an acute MI without HF. The aim is to randomize 3570 patients 1:1 to beta-blocker therapy or no beta-blocker therapy. A study overview is presented in Fig. 1. The expected enrolment period is 2 years and the anticipated duration of the trial is approximately 4 years with subsequent publication of trial results. The trial is designed to achieve a pre-specified number of events. Study patients will be followed for all clinical endpoints until the end of the trial.

### Eligibility criteria

Study patients must be at least 18 years old, have suffered a first-time or recurrent MI within the previous 14 days, and have an LVEF > 40% estimated by an echocardiographic examination after MI. MI is defined using the criteria developed by the Third Universal Definition of Myocardial Infarction [27]. Indications and contraindications to beta-blocker therapy are all relative and assessed by the attending physician and study nurse. Any indication or contraindication will exclude the patient from the trial. Patients treated with a beta-blocker prior to the MI or initiated during the current hospital admission are also eligible (e.g., if the indication is based on a prior MI). Eligible patients may be randomized prior to, after, or without a coronary angiography. Patients will be randomized after informed consent has been obtained by the study personal. A full list of inclusion and exclusion criteria is given in Table 1.

**Table 1:** Inclusion and exclusion criteria

**Inclusion criteria**	
• Myocardial infarction within 14 days according to the Universal ESC definition of MI: Detection of a rise and/or fall of cardiac biomarker values with at least one value above the 99th percentile upper reference limit and with at least one of the following:	
• Symptoms of ischemia	
• New or presumed new significant ST-segment–T wave changes or new left bundle branch block	
• Development of pathological Q waves in the ECG	
• Imaging evidence of new loss of viable myocardium or new regional wall motion abnormality	
• Age > 18 years	
• LVEF > 40% by any imaging technique during hospitalization	
**Exclusion criteria**	
• Any condition that requires beta-blocker treatment according to the treating physician, including but not limited to:	
• Beta-blocker-treated arrhythmias	
• Beta-blocker-treated hypertension	
• Reduced left ventricular ejection fraction	
• Cardiomyopathies	
• Any condition in which beta-blocker treatment is contraindicated according to the treating physician, including but not limited to:	
• Hypotension	
• Brady-arrhythmias	
• Severe peripheral artery disease	
• History of not able to tolerate beta-blocker therapy	
• Severe chronic obstructive pulmonary disease or asthma	
• Severe valvular heart disease	
• Any condition (i.e., dementia) that could lead to increased risk for the patient when treated with beta-blockers	
• Clinical evidence of heart failure at the time of randomization	
• Lack of signed informed consent and expected cooperation during follow-up	
• Pregnancy or of child bearing age not using safe contraception throughout the study period	

### Medical treatment and adherence

After informed consent has been collected, study patients will be randomized 1:1 to receive either beta-blocker therapy or standard treatment without beta-blockers. After randomization both treatment groups will receive standard care after MI and no trial-specific visits are planned. Type and dosage of beta-blocker are at the discretion of the treating physician. The following generic beta-blockers and dosages are recommended: metoprolol succinate up to a total dosage of 200 mg daily, bisoprolol up to a total dosage of 10 mg daily, carvedilol up to a total dosage of 50 mg daily, and nebivolol up to a total dosage of 10 mg daily. It is recommended to titrate to the highest dosage tolerated. The patient will cover cost of medicine.

The patient’s general practitioner will be informed of the trial in the discharge letter and will be encouraged to maintain the patient in the group to which he or she was randomized. Randomized patients will receive a pocket card containing a brief explanation of the trial, information on the randomization group, and contact information on the local responsible investigator. Information on compliance, dosage, and cross-over will be monitored through e-questionnaires to the patients every 3 months. In addition, concomitant medication at the time of randomization and throughout the study period will be ascertained through linkage to the Danish Prescription Registry at study end.

### Data collection and study endpoints

#### Primary endpoints and their rationale

The primary endpoint is a composite of all-cause mortality, recurrent MI, acute decompensated HF, unstable angina pectoris, or stroke. The primary composite endpoint was chosen for several reasons: a composite endpoint addresses a multiplicity of concerns and an integrated effect of beta-blocker therapy is most relevant when considered for later recommendations. All components in the composite endpoint are affected by the use of beta-blockers based on evidence from previous trials. The landmark trials found a mortality benefit from betablocker treatment [1–3] and a relative reduction of reinfarction [2, 3] and no association was seen between beta-blocker treatment and risk of incident HF [28]. In contrast, an increased risk of HF with beta-blocker treatment is supported by a meta-analysis of 12 randomized controlled trials from the reperfusion era [11]. Stroke has been included as a component of the composite endpoint as poorer blood pressure control can potentially occur in the non-beta-blocker group, increasing the risk of stroke. Further, use of a single primary outcome would have resulted in an unfeasible sample size. The primary composite endpoint will be assessed through locally reported and adjudicated endpoints. Adjudication will be performed through review of hospital records, applying endpoint definitions following the objective criteria in the 2017 Cardiovascular and Stroke Endpoint Definition for Clinical Trials [29]. At study end data will be supplemented by linkage to the Danish administrative and clinical registers. The use of registers will ensure no outcome data are missed.

#### Secondary endpoints and their rationale

The secondary endpoints are listed in Table 2 and a schedule of enrolment, interventions, and assessments is presented in Table 3. Among these is the effect of long-term beta-blocker therapy on quality of life as studies investigating the side effects of beta-blocker treatment have given disparate results. Previous trials have demonstrated that beta-blocker therapy may increase fatigue, institute sexual dysfunction, and lead to sleep disorders, gastrointestinal discomfort, cold hands, and feet, bronchospasm, and depressive symptoms [2]. In a recent systemic review of 15 placebo-controlled studies, however, no significant increase in depressive symptoms was found with beta-blocker treatment and only a small increased risk of fatigue and sexual dysfunction were seen [30].

**Table 2:** Primary and secondary endpoints

**Primary endpoints**	
• A composite of all-cause mortality and hospitalization for recurrent non-fatal myocardial infarction, unstable angina pectoris, stroke, or acute decompensated heart failure	
**Secondary endpoints**	
• Each of the components of the primary outcome	
• Cardiovascular mortality	
• Atrial fibrillation and atrial flutter	
• Cardiac arrest	
• Ventricular arrhythmias	
• Stable angina pectoris	
• Bradycardia, syncope, or need for pacemaker	
• Hospitalization for asthma and chronic obstructive pulmonary disorder	
• Hospitalization for diabetes (new onset and dysregulation)	
• Hospitalization for dysregulated blood pressure	
• Peripheral artery disease	
• Blood pressure control^a^	
• Exercise capacity^a^	
*Patient reported outcomes (PRO) regarding*:	
• Health-related quality of life, depression, sexual dysfunction, angina burden following MI, and sleep disorders	

^a^Available for patients attending cardiac rehabilitation

**Table 3:** Schedule of enrolment, interventions, and assessments

Time and assessment	Enrolment	Treatment period following randomization				Study end
	At randomization	Every 3 months	3 months	1 year	2 year	
Eligibility screen, informed consent, allocation to treatment group, and collection of baseline data^a^	x					
Self-reported questionnaires on quality of life and symptom burden^b^	x		x	x	x	
Risk factor control and benefit from cardiac rehabilitation^c^						x
Adherence to treatment group^d^		x				x
Information on hospital admissions^e^		x				x
Endpoints from registry data						x

^a^ Data collected during hospital admission or at subsequent visit

^b^ The following e-questionnaires on patient-reported outcomes will be administered: EQ5D, HADS, IIEF/FSFI (short versions), Bergen Insomnia Scale, NYHA, CCS, and SAQ (for those reporting symptoms of angina)

^c^ Data on blood pressure, serum lipids, diabetes, HbA1c, and VO2peak before and after rehabilitation through registry linkage to the Danish Heart Rehabilitation Database. The data are available for patients participating in cardiac rehabilitation

^d^ Self-reported continued adherence to treatment group will be gathered every 3 months. By linkage to the National Prescription Register at study end, adherence to treatment group as well as other medications will be assessed

^e^ Every 3 months the patient’s vital status and hospitalizations will be investigated through self-reported questionnaires or review of electronic health records. All hospital admissions will be evaluated for a possible relationship with treatment group. At study end hospital admission or death from a primary or secondary endpoint will be ascertained from the Danish National Patient Register

Despite complete revascularization, a significant proportion of patients continues to have angina, which affects quality of life and is associated with anxiety and depression [31, 32]. Beta-blockers are first-line treatment of angina pectoris [33], but whether routine treatment will improve symptoms and quality of life in a contemporary post-MI population is unknown.

Patient reported outcomes will be collected at baseline prior to randomization and after 3, 12, and 24 months through questionnaires (electronic or paper). The following patient reported outcomes will be assessed: quality of life (EuroQol-5 Domain (EQ5D) [34]), anxiety and depression (Hospital Anxiety and Depression Scale (HADS) [35]), sexual dysfunction (short versions of International Index of Erectile Function (IIEF) [36] and Female Sexual Function Index (FSFI) [37]), sleep disorders (Bergen insomnia scale [38]), and symptom-burden after MI (New York Heart Association classification (NYHA), Canadian Cardiovascular Society grading of angina pectoris (CCS), Seattle Angina Questionnaire (SAQ) [39]).

Beta-blocker therapy is no longer first line treatment for hypertension; however, routine use after MI may give better blood pressure control than individual uptitration and may be a part of a beneficial effect of beta-blockers. Hence, blood pressure control has been chosen as a secondary endpoint. The use of beta-blockers in patients with diabetes may reduce insulin sensitivity [40], increase plasma glucose, mask hypoglycaemic symptoms, or increase the risk of new onset diabetes [41–43], but limited data are available. Furthermore, beta-blockers are suspected to limit VO_2_-peak, an objective indicator of physical capacity and a predictor of prognosis. DANBLOCK will ascertain data from the Danish Cardiac Rehabilitation Database (DHRD), a nationwide rehabilitation database of adults treated for ischemic cardiac disease in a Danish hospital. The database contains several quality indicators, including, but not limited to, LVEF, VO_2−_peak, incidence and control of diabetes, blood pressure control, cholesterol levels, and adherence to and dosage of beta-blockers, statins, and platelet inhibitors.

### Study organization, safety, and monitoring

DANBLOCK is a nationwide Danish trial endorsed by the Danish Society of Cardiology. The trial is investigator-initiated and currently 26 of the 29 departments of cardiology in Denmark are participating. A steering committee is responsible for conducting the trial and reporting of the trial results.

Every 3 months patient health records will be reviewed for clinical endpoints and hospitalization with possible causal relation to the treatment or non-treatment with beta-blockers.

Three months after the first patient is randomized and every 6 months thereafter a Data Safety Monitoring Board (DSMB) consisting of two senior cardiologists and one experienced trial statistician will overview safety. In case of an imbalance in event rates between the treatment arms or if the DSMB is of the conviction that the risk to current and future trial patients outweighs the potential impact of continuing the trial, the DSMB will recommend the steering committee to discontinue the trial. DSMB members are independent and not otherwise involved in the trial.

The trial may be discontinued for futility if the inclusion rate is lower than expected and the trial cannot reach the needed number of patients within a reasonable time frame and in case of excessive cross-over. The steering committee will make any decisions of discontinuation.

### Patient and public involvement

Patient groups are involved as research partners to ensure that the trial focuses on issues relevant to the patients and the public. Prior to enrolment of the first patient a patient expert group has been established with members from different backgrounds reflecting patients eligible for the trial. The group has met to share their experiences with beta-blocker treatment and to review and develop patient material, questionnaires, and e-mails to the patients included in the trial. Further meetings of the group will be undertaken during the study period to achieve adequate participant enrolment and ensure continued patient involvement in the process of interpretation and dissemination of results.

### Ongoing beta-blocker trials

In Norway and Sweden trials with comparable inclusion criteria and endpoints are ongoing: The Swedish Randomized Evaluation of Decreased Usage of betablockers after myocardial infarction in the SWEDEHEART registry (REDUCE-SWEDEHEART, NCT03278509) and the Norwegian BEtablocker Treatment After acute Myocardial Infarction in revascularized patients without reduced left ventricular ejection fraction (BETAMI, NCT03646357 [44]). Both trials are designed as superiority trials and aim to include 7000 and 10,000 patients, respectively. Table 4 shows the characteristics of the three Scandinavian studies. The trials primarily differ in terms of the primary endpoint and the LVEF cutoff value.

**Table 4:** Characteristics of DANBLOCK, REDUCE-SWEDEHEART, and BETAMI

	DANBLOCK	REDUCE-SWEDEHEART	BETAMI
**Inclusion criteria**			
Age	≥ 18 years	≥ 18 years	≥ 18 years
MI definition	The universal definition of MI	The universal definition of MI (type 1)	The universal definition of MI (type 1)
Randomized prior to	Day 14 after MI	Day 7 after MI	Not specified
Revascularization	No criteria for revascularization	Obstructive coronary artery disease documented by coronary angiography	PCI or thrombolysis during hospitalization
LVEF cutoff value	> 40%	≥ 50%	≥ 40%
**Exclusion criteria**	Any medical condition where beta-blocker therapy is indicated or contraindicated according to the treating physician	Any medical condition where beta-blocker therapy is indicated or contraindicated according to the treating physician	Any medical condition where beta-blocker therapy is indicated or contraindicated according to the treating physician
**Primary endpoints**	A composite of all-cause mortality and hospitalization for recurrent non-fatal MI, unstable angina pectoris, stroke, and acute decompensated heart failure	Time to the composite of death of any cause or MI	Time to the composite of all cause mortality or non-fatal MI
**Expected number of randomized patients**	3570	7000	10,000
**Expected number of events**	900	944	794
**Expected number of events to contribute to a pooled meta-analysis**	632	944	794
**Individual study power if true HR was 1.2**	0.619	0.797	0.732

Furthermore, a Spanish–Italian trial, REBOOT, is ongoing. REBOOT is randomizing patients after MI with a LVEF > 40% to either beta-blocker therapy or standard treatment without beta-blockers (NCT 03596385). The primary outcome of REBOOT is the 3-year incidence of a composite of all-cause mortality, reinfarction, and HF admission.

### Statistical considerations

The trial is designed to reach a pre-specified number of events and patients will be followed for all clinical endpoints until the end of the trial. Loss to follow-up is considered negligible due to almost complete coverage by the national registers. We must accumulate 900 events to be able to detect an effect with a power of 80% and a hazard ratio of 1.2 for the non-treated group compared with the treated with regard to the primary endpoint. Based on data from the Danish nationwide registry from 2010 to 2015, 9100 patients in Denmark suffer a first-time or recurrent MI annually and the monthly risk of the primary composite endpoint is 1.62%. Some patients will not be eligible to participate in the trial or will not wish to participate; hence, it is estimated that 1785 patients annually will be eligible and willing to sign informed consent. It is expected that a 2-year recruitment period and subsequent follow-up of 3570 patients with aggregated events will yield the desired 900 events. In case of a lower event rate, the inclusion period and the follow-up period will be prolonged and a greater number of patients will be included. Patients will be randomized 1:1 and stratified by LVEF 41–49% and ≥ 50% via a central computerized system. Analysis will be performed using the intention-to-treat principle with a significance level of 5%. In addition, a per-protocol analysis will be carried out. Withdrawal of consent is not expected to be an issue throughout the study period.

#### Combined analysis of the Scandinavian beta-blocker trials

**Fig. 2:**
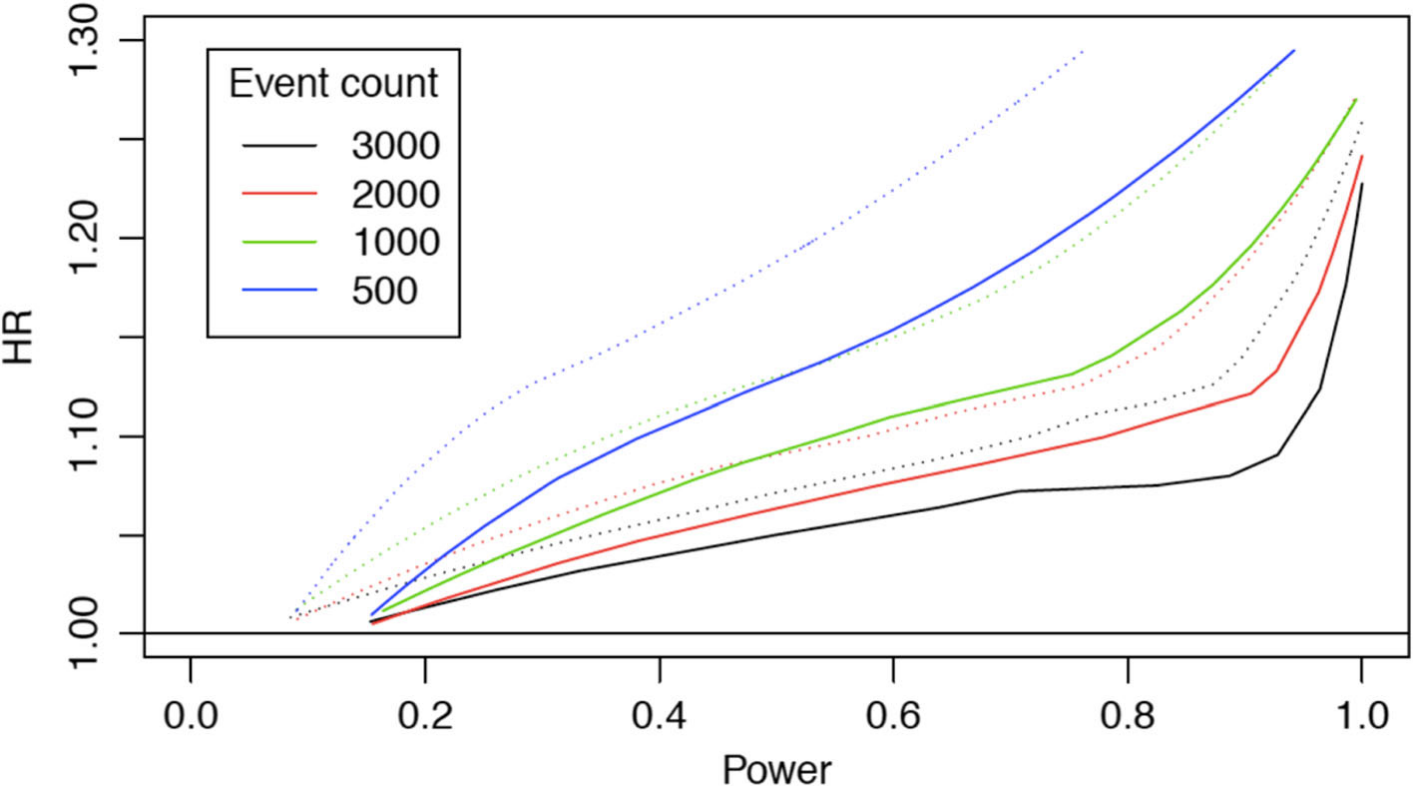
Minimal detectable difference between groups (HR) and statistical power in the planned meta-analyses of the three Scandinavian trials. For comparison the greater statistical power in a single multicenter trial with the same number of events is also depicted. The *solid line* indicates analysis based on data from a single multicenter trial; the *dashed line* indicates meta-analysis performed with a random-effects model

An adequately powered trial is needed to resolve the question of long-term beta-blocker therapy after MI and allow for subgroup analysis, which is possible with a meta-analysis based on data from the three Scandinavian studies. Hence, BETAMI, REDUCE-SWEDEHEART, and DANBLOCK will collaborate on a combined individual patient data meta-analysis when follow-up has ended and data are available. By joining the three studies more than 20,000 patients will be eligible for subgroup analysis, including midrange LVEF, sex, age, treatment dosage, and type of MI. Collating three nationwide trials with pragmatic inclusion criteria, a diverse patient population will increase generalizability of the results. To be able to conduct the meta-analysis, DANBLOCK will report the effect measure as time to the composite of death from any cause or MI, similarly to REDUCE-SWEDEHEART and BETAMI. Differences in inclusion and exclusion criteria are present (Table 4) but arguably less than typically seen in comparable meta-analyses. With a combined expected number of events of 2370 and using a standard random effects meta-analysis, the meta-analysis will have an overall power of 0.993 to detect a HR of 1.2 using a two-sided test at 5% significance level. Figure 2 compares the power for different sample sizes, expressed as expected number of events, and assumed effect size if data originate from a multicenter trial and the planned random effects meta-analysis. When varying the total sample size, the ratios between the studies’ sample sizes are kept fixed as in the actual Scandinavian sample sizes. As a benchmark, Fig. 2 also reports the power obtained if the same number of patients had been included in a single large trial.

## Trial status

The first patient was enrolled December 18th 2018. Recruitment is expected to be complete in the 2021 and end of follow-up in 2022. Results will be available in 2023. The full protocol is available to the public online. The final version is 1.7 November 26th 2018. In case of important protocol modifications, changes will be communicated by e-mail and/or letter to relevant parties (investigators, participants, etc.).

## Discussion

DANBLOCK will add important scientific evidence for the efficacy of beta-blocker treatment following MI in patients without HF. The trial is designed as a prospective, randomized, controlled, open-label, non-blinded endpoint clinical trial. The design is pragmatic and similar to standard clinical practice, which increases the generalizability of its conclusions to routine medical care [45]. It is possible to randomize patients who have not undergone coronary angiography and patients who were treated with a beta-blocker prior to the MI. This will allow for a greater number of patients being randomized, reducing the total inclusion time. A limitation of the selected design is the lack of a placebo group with particular relevance to affect patient-reported outcomes. To minimize this effect patients will complete the baseline questionnaire before randomization.

### LVEF cutoff value

Beta-blocker therapy has a mortality benefit in patients with HF [46]. Consequently, patients with clinical evidence of HF or LVEF ≤ 40% are excluded from the trial. The 2016 ESC guideline introduced the classification of LVEF into preserved (≥ 50%), midrange (40–49%), and reduced LVEF (< 40%) [47]. Accordingly, few data exist on midrange LVEF. Whether or not to include patients with midrange LVEF was considered in depth. A meta-analysis of beta-blockers for HF with reduced, mid-range, and preserved ejection fraction demonstrated no evidence of benefit of beta-blockers for all-cause mortality when LVEF was > 40% in sinus rhythm [48]. The ongoing trials DANBLOCK, BETAMI, and REBOOT and the published CAPITAL-RCT [12] all include patients with midrange LVEF. Hopefully, these trials will resolve whether beta-blockers are beneficial in this group of patients.

### Dosage and duration of beta-blocker therapy

The appropriate length of beta-blocker therapy after MI remains unclear because this issue has not been addressed in any randomized controlled trial. In a systematic review of propensity score matched observational studies and regression cohort studies of long term beta-blocker treatment (> 1 year, median: 3 years) in post-MI patients without HF, the majority of studies failed to identify a benefit on mortality or major cardiovascular events with long-term beta-blocker therapy [49]. DANBLOCK, BETAMI, REBOOT, and REDUCE-SWEDEHEART will investigate the long-term efficacy with an expected treatment duration of 2–4 years.

The dosages used in contemporary clinical practice are lower than dosages used in previous randomized clinical trials [50] and may contribute to the lack of a mortality benefit seen in some observational studies. Interestingly, register-based studies suggest that an increased survival might not be present in patients treated with dosages approximating those from the early beta-blocker trials compared with a lower dosage [51]. A register-based study showed similar rates of major adverse cardiac events among patients receiving low-dose and high-dose beta-blocker therapy (respectively ≤ 25% and ≥ 50% of an equivalent daily dose of 200 mg metoprolol) during 6–24 months after acute coronary syndrome (hazard ratio 1.03, 95% CI 0.70–1.50) [52]. In DANBLOCK the type and dosage of beta-blocker are at the treating cardiologist’s choice. It is recommended to achieve the highest dosage tolerated. In the discharge letter it is recommended to increase the dosage at cardiac rehabilitation and subsequent follow-up.

### Utility of withdrawal studies

Since the 1980s the proportion of adults over 65 years who are prescribed five or more medications has tripled [53]. The effectiveness of secondary prevention depends on patient adherence and patients may only adhere to half of the treatment prescribed [54]. In studies investigating adherence to secondary preventive therapies after MI, only half of the population were adherent to ACE inhibitors/ARBs, beta-blockers, and statins [23]. In the same study, mortality was equal between a group adherent to all three medications and a group only adherent to ACE inhibitors/ARBs and statins. Baseline characteristic were equal in the two groups. These findings demonstrate the need for withdrawal studies to ensure that the patients are adherent to the most efficacious treatment. It is in line with the ESC’s suggestion to improve research on secondary prevention after acute coronary syndromes to replace old evidence-based treatments instead of incrementally adding new ones [55].

## Conclusion

DANBLOCK will add important scientific evidence for the efficacy of beta-blocker treatment following MI in patients without HF and reduced LVEF. The collaborative effort of the three Scandinavian studies will have the potential of changing guidelines and clinical practice to the benefit of these MI patients.

## Supplementary information

**Additional file 1.** World Health Organization trial registration data set.

**Additional file 2.** Overview of meta-analyses regarding beta-blocker therapy after myocardial infarction.

## Data Availability

Data sharing is not applicable to this article. No datasets have been generated yet as the study is still ongoing.

## Abbreviations

ACC/AHA: American College of Cardiology/American Heart Association
BETAMI: BEtablocker Treatment After acute Myocardial Infarction in revascularized patients without reduced left ventricular ejection fraction
CI: Confidence interval
DANBLOCK: Danish trial of beta-blocker treatment after myocardial infarction without reduced ejection fraction
DHRD: Danish Cardiac Rehabilitation Database
DSMB: Data Safety Monitoring Board
ESC: European Society of Cardiology
HF: Heart failure
LOE: Level of evidence
LVEF: Left ventricular ejection fraction
MI: Myocardial infarction
NSTEMI: Non-ST-elevation myocardial infarction
PCI: Primary percutaneous coronary intervention
REDUCE-SWEDEHEART: Swedish Randomized Evaluation of Decreased Usage of betablockers after myocardial infarction in the SWEDEHEART registry
STEMI: ST-elevation myocardial infarction
