# Supplementary material for: Design and rationale of the Danish trial of beta-blocker treatment after myocardial infarction without reduced ejection fraction: study protocol for a randomized controlled trial
Source: Trials. 2020 May 23;21:415. doi: 10.1186/s13063-020-4214-6 (PMC7245032; doi:10.1186/s13063-020-4214-6)
Supplement: Supplementary file 1 — Additional file 1. World Health Organization trial registration data set. [file 13063_2020_4214_MOESM1_ESM.docx]

### Additional file 1: World Health Organization Trial Registration Data Set.

| **Data category** | **Information** |
| --- | --- |
| Primary registry and trial identifying number and date of registration in primary registry | Clinicaltrials.gov, NCT03778554, registered on 18 December 2018  European Clinical Trials Database, 2018-000590-75, registered on 28 September 2018 |
| Primary sponsor | Professor Eva Prescott ([eva.irene.bossano.prescott@regionh.dk](mailto:eva.irene.bossano.prescott@regionh.dk)), Department of Cardiology, Bispebjerg University Hospital, Denmark |
| Contact for public and scientific queries | Professor Eva Prescott ([eva.irene.bossano.prescott@regionh.dk](mailto:eva.irene.bossano.prescott@regionh.dk)) |
| Scientific title | Danish trial of beta-blocker treatment after myocardial infarction without reduced ejection fraction: DANBLOCK |
| Countries of recruitment | Denmark |
| Health condition(s) or problem(s) studied | Long term prognosis after myocardial infarction |
| Intervention | Beta-blocker therapy or standard treatment without beta-blocker therapy |
| Key inclusion and exclusion criteria | *Inclusion Criteria:*  Left ventricular ejection fraction > 40%  Myocardial infarction (MI) within previous two weeks  The diagnosis of acute MI must meet the Universal European Society of Cardiology (ESC) definition of MI  *Exclusion Criteria:*  Clinical evidence of heart failure at the time of discharge  Pregnancy or of child bearing age not using safe anticonception throughout the study period  Lack of signed informed consent and expected cooperation during follow-up  Any medical condition where beta-blocker treatment is indicated according to the treating physician |
| Study type | Prospective, randomized, controlled, open-label, non-blinded endpoint clinical trial |
| Date of first enrolment | December 18, 2018 |
| Target sample size | 3570 |
| Recruitment status | Recruiting |
| Primary outcomes | A composite of all-cause mortality, recurrent MI, acute decompensated heart failure, unstable angina pectoris, or stroke. |
