# Supplementary material for: Design and rationale of the Danish trial of beta-blocker treatment after myocardial infarction without reduced ejection fraction: study protocol for a randomized controlled trial
Source: Trials. 2020 May 23;21:415. doi: 10.1186/s13063-020-4214-6 (PMC7245032; doi:10.1186/s13063-020-4214-6)
Supplement: Supplementary file 2 — Additional file 2. Overview of meta-analyses regarding beta-blocker therapy after myocardial infarction. [file 13063_2020_4214_MOESM2_ESM.docx]

### Additional file 2: Overview of meta-analyses regarding beta-blocker therapy after myocardial infarction

| Title, author, year | Inclusion criteria  *Type of studies*  *Sample size*  *STEMI/NSTEMI/MI*  *Follow up duration*  *Reperfusion status*  *Other inclusion criteria* | Characteristics of meta-analysis  *Type and number of studies included*  *Number of patients*  *STEMI/NSTEMI/MI*  *Reperfusion status*  *Follow up duration (mean/median, range)*  *Other characteristics of interest* | Outcomes  *Primary outcome*  *-Results*  *Secondary outcomes* | Subgroup analyses  *LVEF*  *Long term follow up*  *Subgroup analysis of particular interest* | Conclusion |
| --- | --- | --- | --- | --- | --- |
| *Title:* Beta-blockade after myocardial infarction: a systematic review and meta regression analysis  *Author*: Freemantle et al  *Year*: 1999 | *Type of studies included:* RCT’s  *Sample size:* NA  *STEMI/NSTEMI/MI:* MI  *Follow up duration:* >1 day  *Reperfusion status:* NA  *Other inclusion criteria:* NA | *Type and number of studies:* 82 RCT’s  *Number of patients:* 54,234  *STEMI/NSTEMI/MI:* MI  *Reperfusion status:* No reperfusion  *Follow up duration:*  *-Mean/Median*: NA  -*Range*: In hospital - 3 years  *Other characteristics:* NA | *Primary outcome:* All-cause mortality  *-Results:* 23% odds of death in long term trials (95% CI 15% - 31%). 4% reduction in the odds of death in short term trials ( -8% - 15%).  *Secondary outcomes:*  -Non­fatal reinfarction  -Withdrawal from treatment | *LVEF*: No subgroup analysis for preserved LVEF  *Long term follow up*: Long term treatment was defined as 6-48 months. Pooled odds ratio of long-term treatment was 0.77, 95% CI 0.69-0.85. | Beta-blockers are effective in long term secondary prevention after myocardial infarction. |
| *Title:* Clinical Outcomes with beta-blockers for Myocardial Infarction: A Meta-analysis of Randomized Trials  *Author*: Bangalore et al  *Year*: 2014 | *Type of studies included:* RCT’s  *Sample size:* >100  *STEMI/NSTEMI/MI:* MI  *Follow up duration:* NA  *Reperfusion status:* NA  *Other inclusion criteria:* NA | *Type and number of studies:* 60 RCT’s  *Number of patients:* 102,003  *STEMI/NSTEMI/MI:* MI  *Reperfusion status:*  -48 pre-reperfusion studies  -12 reperfusion studies (50% of patients received reperfusion (trombolytics, aspirin, statin))  *Follow up duration:*  -*Mean*: 10 months  -*Range*: In-hospital - 4 years  *Other characteristics:* NA | *Primary outcome:* All-cause mortality  *-Results:* Overall mortality incidence ratio 0.98, 95% CI 0.92-1.05  *Secondary outcomes:* Cardiovascular mortality, sudden death, recurrent myocardial infarction, angina pectoris, heart failure, cardiogenic shock, stroke, and drug discontinuation | *LVEF*: No subgroup analysis for preserved LVEF  *Long term follow up*: Long-term efficacy (>1year) were not investigated in the reperfusion era.  *Subgroup analysis of particular interest:* Cardiovascular mortality, sudden death, recurrent myocardial infarction, angina pectoris, heart failure, cardiogenic shock, stroke, and drug discontinuation in acute and post-MI trials. Analysis stratified by reperfusion status | Beta-blockers have no mortality benefit but reduce recurrent MI and angina (short-term) at the expense of increase in HF, cardiogenic shock, and drug discontinuation in the reperfusion era. |
| *Title:* Meta-Analysis of Relation Between Oral β-Blocker Therapy and Outcomes in Patients with Acute Myocardial Infarction Who Underwent Percutaneous Coronary Intervention.  *Author*: Huang et al  *Year*: 2015 | *Type of studies included:* NA  *Sample size:* >100  *STEMI/NSTEMI/MI:* MI  *Follow up duration:* >3 months  *Reperfusion status:* Treated with PCI  *Other inclusion criteria:* Oral beta-blocker treatment | *Type and number of studies:* 10 observational studies  *Number of patients:* 40,873  *STEMI/NSTEMI/MI:* MI  *Reperfusion status:* PCI  *Follow up duration:* NA  -*Mean/Median*: NA  *-Range*: 1/2 - 4 years  *Other characteristics:* NA | *Primary outcome:* All-cause mortality  *-Results:* Adjusted hazard ratio for all-cause mortality 0.76, 95% CI 0.62-0.94  *Secondary outcomes:*  -Cardiac death  -Myocardial infarction  -Heart failure admission | *LVEF*: All-cause mortality in patients with preserved LVEF: relative risk 0.76, 95% CI 0.59-1.07.  *Long term follow up*: Subgroup analysis with follow-up <1 and >1 year. Efficacy of beta-blocker treatment >1 year after incident MI: relative risk 0.76, 95% CI 0.59-0.98. | The potential benefit of beta-blockers in preventing all-cause death was not similar in all population but was restricted to those with reduced ejection fraction, with low use proportion of other secondary prevention drugs or with none ST-segment elevation myocardial infarction. The association between the use of beta-blockers and improved survival rate was significant in <1-year follow-up duration. Rates of cardiac death, myocardial infarction, and heart failure readmission in patients using beta-blockers were not significantly different from those in patients without beta-blocker therapy. In conclusion, there is lack of evidence to support routine use of b-blockers in all patients with AMI who underwent PCI. |
| *Title:* Does Oral Beta-Blocker Therapy Improve Long-Term Survival in ST-Segment Elevation Myocardial Infarction with Preserved Systolic Function? A Meta-Analysis  *Author*: Misumida et al  *Year*: 2016 | *Type of studies included:* RCT’s/observational studies  *Sample size:* NA  *STEMI/NSTEMI/MI:* STEMI  *Follow up duration:* >6 months  *Reperfusion status:* Treated with PCI  *Other inclusion criteria:*  -Preserved LVEF  -Available adjusted HR for observational studies or HR in propensity score-matched patients | *Type and number of studies:* 7 observational studies  *Number of patients:* 10,857  *STEMI/NSTEMI/MI:* STEMI  *Reperfusion status:* PCI  *Follow up duration:*  *-Mean/median:* NA  *-Range:* 6 months - 5.2 years  *Other characteristics:* NA | *Primary outcome:* All-cause mortality  *-Results:* Combined hazard ratio for all-cause mortality 0.79, 95% CI 0.65-0.97 | *LVEF*: Primary analysis of LVEF>40%  *Long term follow up*: Primary analysis with follow up duration >6 months | In patients with STEMI undergoing primary PCI, who have preserved LVEF oral beta-blocker treatment was associated with decreased all-cause mortality. |
| *Title:* Meta-Analysis Comparing Metoprolol and Carvedilol on Mortality Benefits in Patients with Acute Myocardial Infarction  *Author*: Li et al  *Year*: 2017 | *Type of studies included:* RCT’s  *Sample size:* NA  *STEMI/NSTEMI/MI:* MI  *Follow up duration:* NA  *Reperfusion status:* NA  *Other inclusion criteria:* Comparison of carvedilol or metoprolol with placebo or RCTs directly comparing carvedilol with metoprolol. | *Type and number of studies:* 12 RCT’s  *Number of patients:* 61,081  *STEMI/NSTEMI/MI:* MI  *Reperfusion status:* Except for 5 trials not reporting the baseline treatments, all trials adopted guideline-recommended standard therapies for MI, with only 1 trial adopting primary PCI  *Follow up duration:*  *-Mean/median*: NA  *-Range:* 0,5 - 40 months  *Other characteristics:* NA | *Primary outcome:* All-cause death  *-Results:* Carvedilol vs placebo: Risk ratio for all-cause death was 0.73, 95% credible interval 0.47-1.10  Metoprolol vs placebo: Risk ratio 0.86, 95% credible interval 0.70-1.00  *Secondary outcomes:* A composite cardiovascular events (cardiovascular death, nonfatal re-infarction, and nonfatal stroke), sudden death, cardiovascular death, re-infarction, revascularization, rehospitalization, ventricular arrhythmia, drug discontinuation for all reasons except for death. | *LVEF*: No subgroup analysis for preserved LVEF.  *Long term follow up*: No subgroup analysis of long-term efficacy  *Other subgroup analysis of particular interest*: Compared with placebo, carvedilol and metoprolol reduced composite cardiovascular events in patients with MI (risk ratio 0.63, 95% credible interval 0.41-0.85 for carvedilol; risk ratio 0.78; 95% credible interval 0.65-0.93 for metoprolol). | Pooled results showed that compared with placebo, carvedilol and metoprolol significantly reduced composite cardiovascular events and re-infarction in patients with MI. However, neither carvedilol nor metoprolol showed significant benefits on all-cause death, cardiovascular death, revascularization, and rehospitalization. Also, no obvious difference was found when comparing carvedilol and metoprolol on primary or secondary outcomes. |
| *Title:* Effect of oral beta-blocker treatment on mortality in contemporary post-myocardial infarction patients: a systematic review and meta-analysis  *Author:* Dahl Aarvik et al  *Year:* 2018 | *Type of studies included:* All study types and sizes  *Sample size:* NA  *STEMI/NSTEMI/MI:* MI  *Follow up duration:* NA  *Reperfusion status:* NA  *Other inclusion criteria:*  -None or only a minority with HF, Killip class over 3 or LVEF<40%.  -Studies published after 1. January 2000 | *Type and number of studies:* 16 observational studies  *Number of patients:* 164,408  *STEMI/NSTEMI/MI:* MI  *Reperfusion status:* 45.9% had PCI/CABG and 72.1% were treated with primary PCI for STEMI.  *Follow up duration:*  *-Median:* 2.7 years  *-Range:* 0.5 - 5.2 years  *Other characteristics:* NA | *Primary outcome:* All-cause mortality  *-Results:* Rate ratio for all-cause mortality was 0.74, 95% CI 0.64–0.85. After controlling for bias the rate ratio was 0.90, 95% CI 0.77–1.04 | *LVEF*: Primary analysis of none or only a minority with HF, Killip class over 3 or LVEF<40%.  *Long term follow up*: Primary analysis with a median follow up of 2.7 years | The results from the meta-analysis of nearly 200,000 patients following AMI of whom only a minority had reduced LVEF and/or clinical signs of HF, provides evidence that the association between beta-blockers and long-term survival is due to small study effect, and that there might not be a significant reduction in the risk of all-cause mortality when controlling for bias. After controlling for bias no mortality effect of beta-blocker therapy was found. |
| *Title:* Beta-blocker therapy reduces mortality in patients with coronary artery disease treated with percutaneous revascularization: a meta-analysis of adjusted results  *Author*: Peyracchia et al  *Year*: 2018 | *Type of studies included:* Studies with multivariate adjustment  *Sample size:* NA  *STEMI/NSTEMI/MI:* NA  *Follow up duration:* >1 year  *Reperfusion status:* Treated with PCI  *Other inclusion criteria:* MI or stable angina | *Type and number of studies:* 26 observational studies  *Number of patients:* 863,335  *STEMI/NSTEMI/MI:* MI  *Reperfusion status:* PCI  *Follow up duration:*  *-Median:* 3 years  *-Range:* 1 - 4.3 years  *Other characteristics:* Patients suffering from MI or stable angina | *Primary outcome:* All-cause death  *-Results:* Risk of all-cause death in patients on beta-blockers after 3 years: odds ratio 0.69, 95% CI 0.66–0.72  *Secondary outcomes:*  -A composite endpoint of all-cause death or MI  -Myocardial infarction | *LVEF*: Subgroup analysis for all cause death in patients with LVEF>40%, odds ratio 0.79, 95% CI 0.69–0.91  *Long term follow up*: After 3 years: odds ratio for all-cause death for patients with MI and stabile angina 0.69, 95% CI 0.66–0.72  *Other subgroup analysis of particular interest*: Subgroup analysis for all cause death in patients with MI (odds ratio 0.60, 95% CI 0.56–0.65) and stable angina patients (odds ratio 0.84, 95% CI 0.78–0.91) | After 3 years, long-term risk of all-cause death was lower in patients on beta-blockers, both for acute coronary syndrome and stable angina patients, independently from ejection fraction. The risk of long-term MACE was lower but not significant for acute coronary syndrome patients treated with beta-blockers as in stable angina. Similarly, risk of myocardial infarction did not differ between patients treated with beta-blockers or without beta-blockers. |
| *Title:* Beta-blockers for heart failure with reduced, mid-range, and preserved ejection fraction: an individual patient-level analysis of double-blind randomized trials  *Author*: Cleland et al  *Year*: 2018 | *Type of studies included:* RCT’s  *Sample size:* Sample > 300  *STEMI/NSTEMI/MI:* NA  *Follow up duration:* > 6 months  *Reperfusion status:* NA  *Other inclusion criteria:* Patients suffering from heart failure | *Type and number of studies:* 11 RCT’s  *Number of patients:* 18,637 (819 patients with LVEF$\geq$40%)  *STEMI/NSTEMI/MI:* 71.8% and 36.1% of patients with 40–49% and $\geq$ 50% had suffered from a prior MI.  *Reperfusion status:* NA  *Follow up duration:*  -*Median*: 1.3 years  -*Interquartile range:* 0.8–1.9 years  *Other characteristics:* Patients with heart-failure | *Primary outcomes:* All-cause mortality and cardiovascular death  *-Results:*  Patients with LVEF<40% in sinus rhythm:  -Kaplan Meier plots for unadjusted all-cause mortality (log rank p<0.001) cardiovascular mortality (log rank p<0.001)  Patients with LVEF40–49% in sinus rhythm:  -All-cause mortality: adjusted HR 0.59, 95% CI 0.34–1.03  -Cardiovascular death: adjusted HR 0.48, 95% CI 0.24–0.97  -Kaplan Meier plots for unadjusted all-cause mortality (log rank p = 0.042) cardiovascular mortality (log rank p = 0.022)  Patients with LVEF$\geq$50% in sinus rhythm:  -All-cause mortality: HR 1.79, 95% CI 0.78–4.10  -Cardiovascular death: 1.77, 95% CI 0.61–5.14  -Kaplan Meier plots for unadjusted all-cause mortality (log rank p = 0.51) cardiovascular mortality (log rank p = 0.57)  *Secondary outcomes:* First cardiovascular hospitalization and the composite of cardiovascular death and cardiovascular hospitalization | *LVEF*: Primary analysis stratified by LVEF<40%, LVEF40–49% and LVEF$\geq$50%  *Long term follow up*: Kaplan Meyer plot performed with treatment duration up to 3 years. | For patients with heart failure in sinus rhythm and LVEF <40%, beta-blockers improve left ventricular systolic function and reduce cardiovascular morbidity and mortality. These benefits also apply to patients with LVEF 40–49%. No benefit was seen in patients with LVEF$\geq$50%, but too few patients have been studied in double-blind RCTs to draw firm conclusions on the efficacy or safety of beta-blockers for HFpEF. |
| Systematic reviews of particular interest | | | | | |
| *Title:* Long-term beta-blocker therapy after myocardial infarction in the reperfusion era: A systematic review  *Author*: Hong and Berry  *Year*: 2018 | *Type of studies included:* RCT’s, regression cohort or propensity score matched studies  *Sample size:* NA  *STEMI/NSTEMI/MI:* MI  *Follow up duration:* > 1 year  *Reperfusion status:* NA  *Other inclusion criteria:*  -LVEF > 30%  -Studies based on data from 1. January 2000 onward | *Type and number of studies:* 8 cohort studies  *Number of patients:* 31,501  *STEMI/NSTEMI/MI:* MI  *Reperfusion status:* NA  *Follow up duration:*  -*Median*: 3 years  -*Range*: 1-5.2 years  *Other characteristics:* NA | Outcomes of interest included all-cause and cardiovascular mortality, nonfatal MI, and nonfatal stroke. | *LVEF*: Primary analysis with LVEF>30%  *Long term duration*: Primary analysis with follow up > 1 year | The majority of the included studies in the systemic review failed to demonstrate a benefit in survival or cardiovascular events with long-term beta-blockers (>1 year) in post-MI patients with normal left ventricular function. |
